# Case Report: Talonavicular joint replacement in congenital talonavicular coalition with individualized 3D-printed prosthesis

**DOI:** 10.3389/fsurg.2026.1865047

**Published:** 2026-10-13

**Authors:** Guimiao Li, Shixin Liu, Chenchen Yuan, Leilei Zhao, Hecheng Zhou, Dehua Liu, Dehang Liu, Gang Ji, Guobin Liu, Zhenhui Sun

**Affiliations:** 1 Department of Orthopedics, The First Hospital of Hebei Medical University, Shijiazhuang, China; 2 Department of Foot and Ankle Surgery, The First Hospital of Hebei Medical University, Shijiazhuang, Hebei, China; 3 Hebei Medical University, Shijiazhuang, China

**Keywords:** 3D-printed prosthesis, flatfoot, motion preservation surgery, talonavicular coalition, talonavicular joint replacement

## Abstract

**Background:**

Talonavicular coalition is a rare type of tarsal coalition that may lead to painful flatfoot and progressive functional impairment. Conventional talonavicular arthrodesis provides symptom relief but sacrifices joint motion. Motion-preserving reconstruction strategies remain largely unexplored.

**Case presentation:**

We report a case of congenital talonavicular coalition accompanied by flatfoot treated with individualized 3D-printed talonavicular joint replacement (TNJR) in a 12-year-old patient. At 24-month follow-up, the patient achieved complete pain relief (VAS 0) and significant functional improvement (AOFAS 98). Talonavicular joint motion was preserved with 15° inversion/eversion and 15° pronation/supination. Radiographic evaluation demonstrated restoration of the medial longitudinal arch and correction of hindfoot valgus. Progressive improvement was observed in the calcaneal pitch, talonavicular coverage, and hindfoot alignment angles, whereas Meary's angle showed a slight increase.

**Conclusion:**

This case demonstrates the feasibility of individualized 3D-printed TNJR as a potential motion-preserving option for selected patients with extensive congenital talonavicular coalition.

## Introduction

Tarsal coalition is defined as an abnormal fibrous, cartilaginous, or osseous connection between the tarsal bones, with a population prevalence of 2%. Tarsal coalition is a common cause of foot and ankle pain in adolescents and an important etiological factor in congenital flatfoot. Talocalcaneal and calcaneonavicular coalitions account for 48.1% and 43.6% of all tarsal coalitions, respectively (1). Talonavicular coalition is relatively rare, accounting for 1%–1.3% of all tarsal coalitions (2, 3). With advancing age, progressive ossification of the coalition and increasing repeated physical activity could lead to symptoms such as foot and ankle pain after activity or prolonged standing (3, 4).

For complex tarsal coalitions, arthrodesis is reserved as a final option. Although fusion can provide effective pain relief, it is associated with several limitations, including irreversible loss of joint motion, increased mechanical stress on adjacent joints that may accelerate secondary degenerative changes, and the risk of nonunion (5–7). Given the patient's young age, extensive talonavicular involvement, and high anticipated functional demands, preserving joint mobility was considered particularly important, prompting the selection of a motion-preserving technique.

Patient-specific reconstruction in foot and ankle surgery has evolved from conventional custom-made implants toward integrated digital planning and additive manufacturing. Advances in CT-based 3D reconstruction, computer-aided design, virtual surgical planning, and patient-specific instrumentation have enabled precise anatomical customization and implant positioning (8–10). Metal 3D printing further facilitates the fabrication of complex geometries and porous or lattice structures that may enhance bone–implant integration and fixation (11, 12). These advances have expanded patient-specific reconstruction from structural replacement to anatomically and functionally oriented solutions.

Within the talonavicular region, patient-specific reconstruction has also been explored. Adams et al. reported a patient-specific 3D-printed navicular replacement while preserving the native talonavicular articulation (13). Giannini et al. described a custom-made total talonavicular prosthesis for post-traumatic talonavicular complex reconstruction, in which the talus and navicular were reconstructed as a single functional unit (14). These pioneering studies provided valuable insights into the feasibility of personalized 3D-printed reconstruction for complex midfoot disorders. However, individualized 3D-printed replacement of both articular surfaces while preserving an independently mobile talonavicular joint remains largely unexplored.

Here, we present a case of individualized 3D-printed TNJR in a patient with congenital talonavicular coalition and flatfoot. This case highlights the feasibility of a patient-specific, motion-preserving approach for complex talonavicular coalition and provides a potential alternative to arthrodesis in selected young patients.

## Case report

### Patient information

This is a 12-year-old male presented with chronic midfoot pain and limited mobility since the age of 6, exacerbated by weight-bearing activities. Conservative management with orthotic devices was attempted but yielded inadequate symptom relief. Restricted talonavicular joint motion and localized tenderness on the right foot were observed on physical examination. The right foot demonstrated flexible flatfoot deformities, calcaneal valgus and forefoot abduction deformity (Figure 1). Range of motion of the hips, knees, and ankles joints was preserved bilaterally. Muscle strength testing revealed grade IV strength of the right tibialis posterior according to the Medical Research Council scale. The patient was diagnosed with congenital talonavicular coalition and flatfoot. Preoperative x-ray and CT confirmed a complete osseous coalition between the talus and navicular, accompanied by radiographic features consistent with talonavicular coalition (Figure 2).

**Figure 1:**
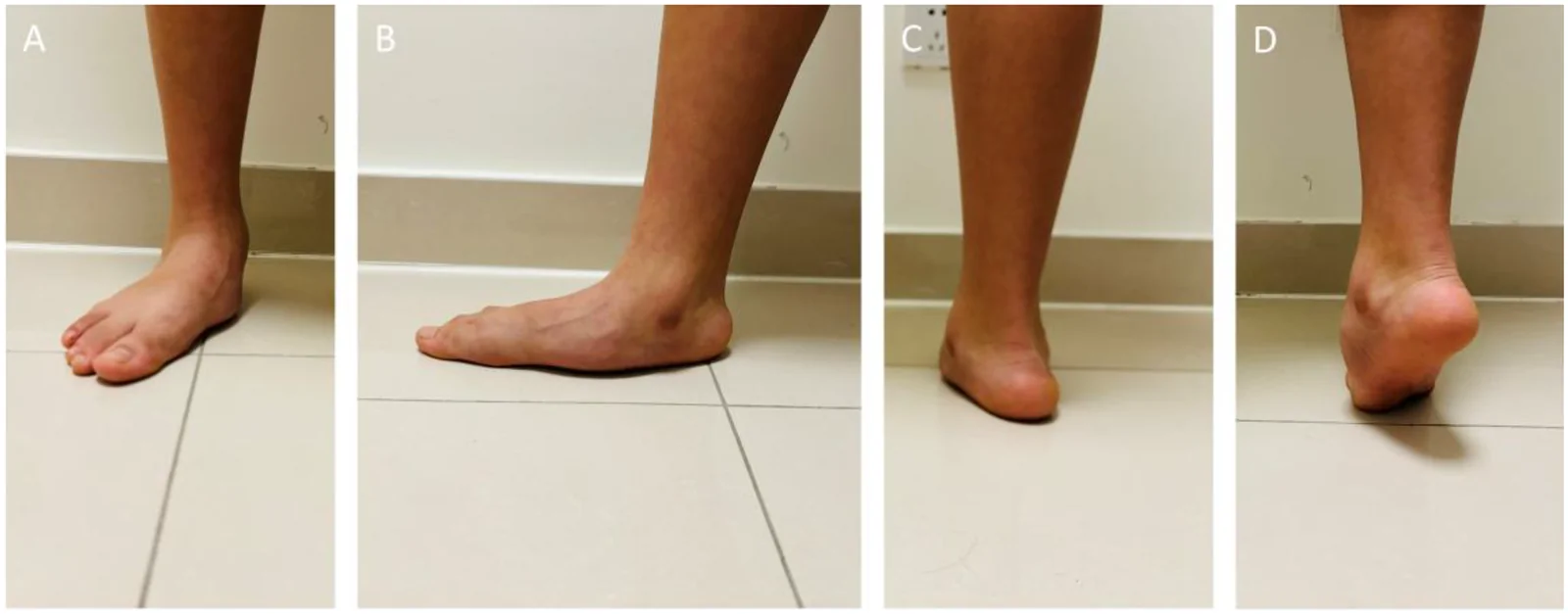
Preoperative photographs of the right foot. **(A,B)** Anteromedial and lateral views demonstrating collapse of the medial longitudinal arch. **(C)** Posterior view showing hindfoot valgus deformity. **(D)** Heel-rise test indicating preserved flexibility of the deformity.

**Figure 2:**
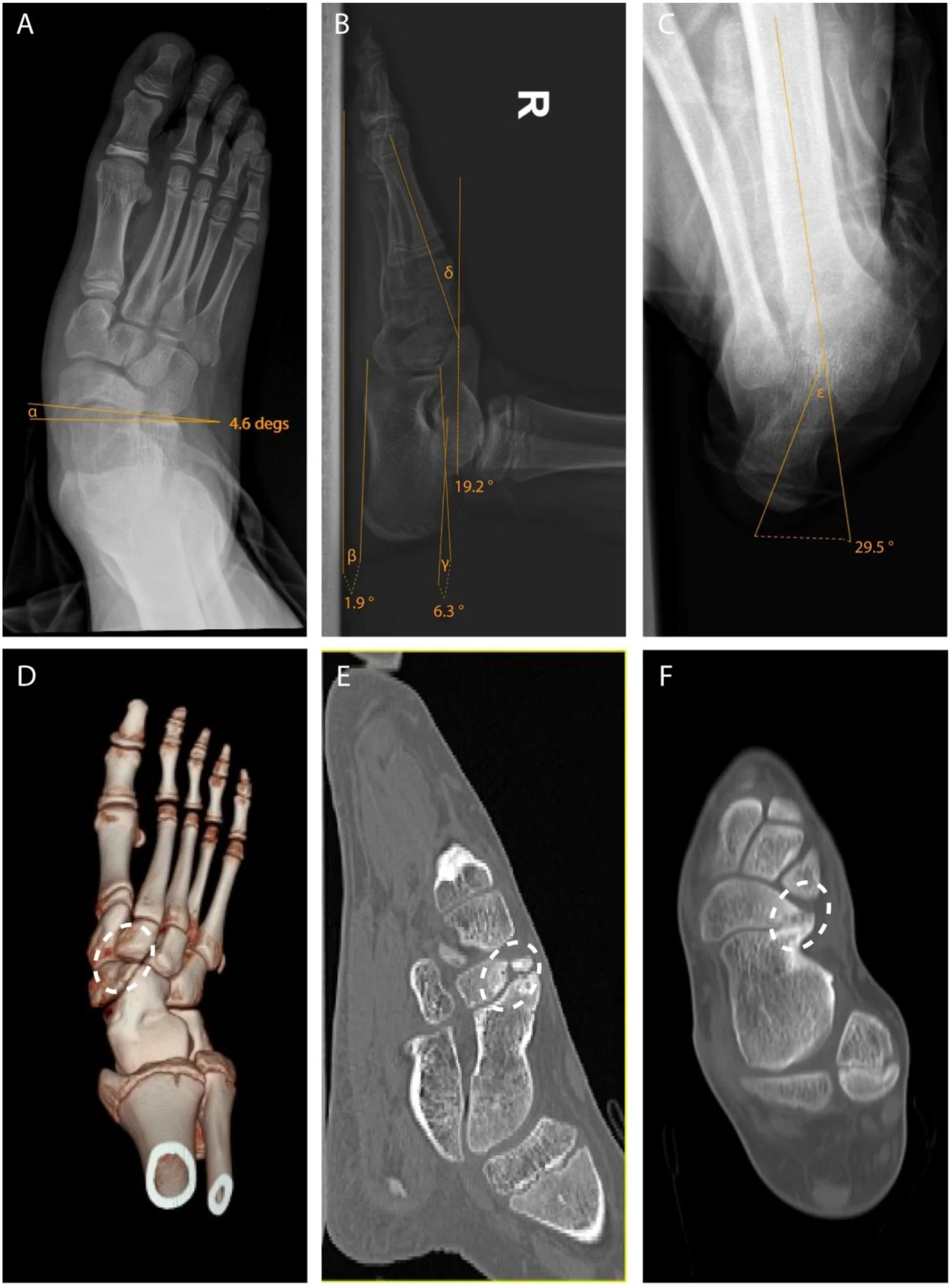
Preoperative radiographic and CT evaluation of the right foot. **(A)** Weight-bearing anteroposterior radiograph *α*:Talonavicular coverage angle 4.6°.**(B)** Weight-bearing lateral radiograph showing collapse of the medial longitudinal arch. *β*:Pitch's angle 1.9°,*γ*:Bohler's angle 6.3°,*δ*: Meary's angle 19.2°.**(C)** Hindfoot alignment view revealing valgus deformity. *ε*: Hindfoot alignment angle 29.5°。**(D)** 3-dimensional CT reconstruction illustrating the talonavicular coalition (dashed circle). **(E)** Sagittal CT image demonstrating coalition morphology and joint involvement. **(F)** Axial CT image confirming the osseous talonavicular coalition.

### Preoperative planning and 3D design

Preoperative high-resolution CT data of the affected foot were imported into Mimics software (Materialise, Belgium) for three-dimensional reconstruction of the talonavicular joint and surrounding bony structures. The implant design was primarily based on the patient-specific anatomy reconstructed from the affected side, while a mirrored model of the contralateral foot was generated for anatomical comparison and optimization of implant morphology. The reconstructed models were used to evaluate the coalition characteristics, determine the optimal resection plane, and develop an individualized surgical plan.

The patient-specific talonavicular prosthesis was designed using Mimics and 3-matic software (Materialise, Belgium). The prosthesis consisted of 3D-printed Ti-6Al-4 V talar and navicular components with a polyethylene bearing insert, forming a mobile-bearing design to restore talonavicular articulation and preserve joint motion. A lattice structure was incorporated into the bone–implant interfaces of the talar and navicular components to facilitate osseointegration (15) (Figure 3). The configuration and distribution of the lattice structures were tailored according to the implant geometry and the patient's anatomical characteristics. The polyethylene bearing insert was manufactured using subtractive machining to achieve precise fitting and smooth articulation (Figure 4). The patient-specific surgical guides required approximately 4 h for digital design and 12 h for printing. The talonavicular prosthesis required approximately 48 h for design, followed by approximately 20 days for manufacturing, quality inspection, and sterilization before implantation. The prosthesis cost 9,272 RMB.

**Figure 3:**
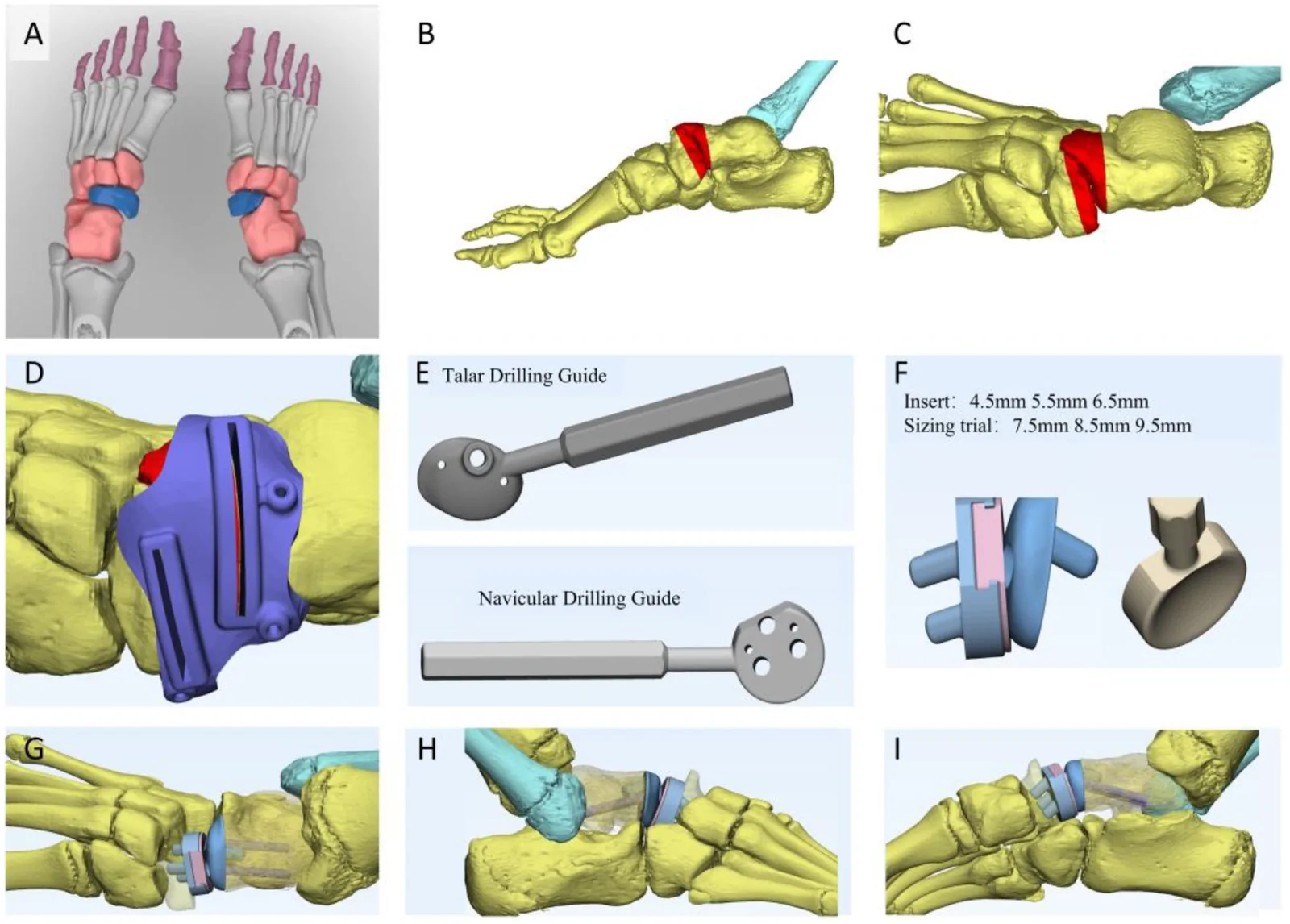
Workflow of individualized prosthesis design and surgical simulation. **(A)** 3D anatomical reconstruction. **(B,C)** Virtual coalition resection and implant positioning. **(D)** Patient-specific osteotomy guide. **(E)** Customized drilling guides. **(F)** Prosthesis design and dimensional planning. **(G–I)** Simulated prosthesis implantation.

**Figure 4:**
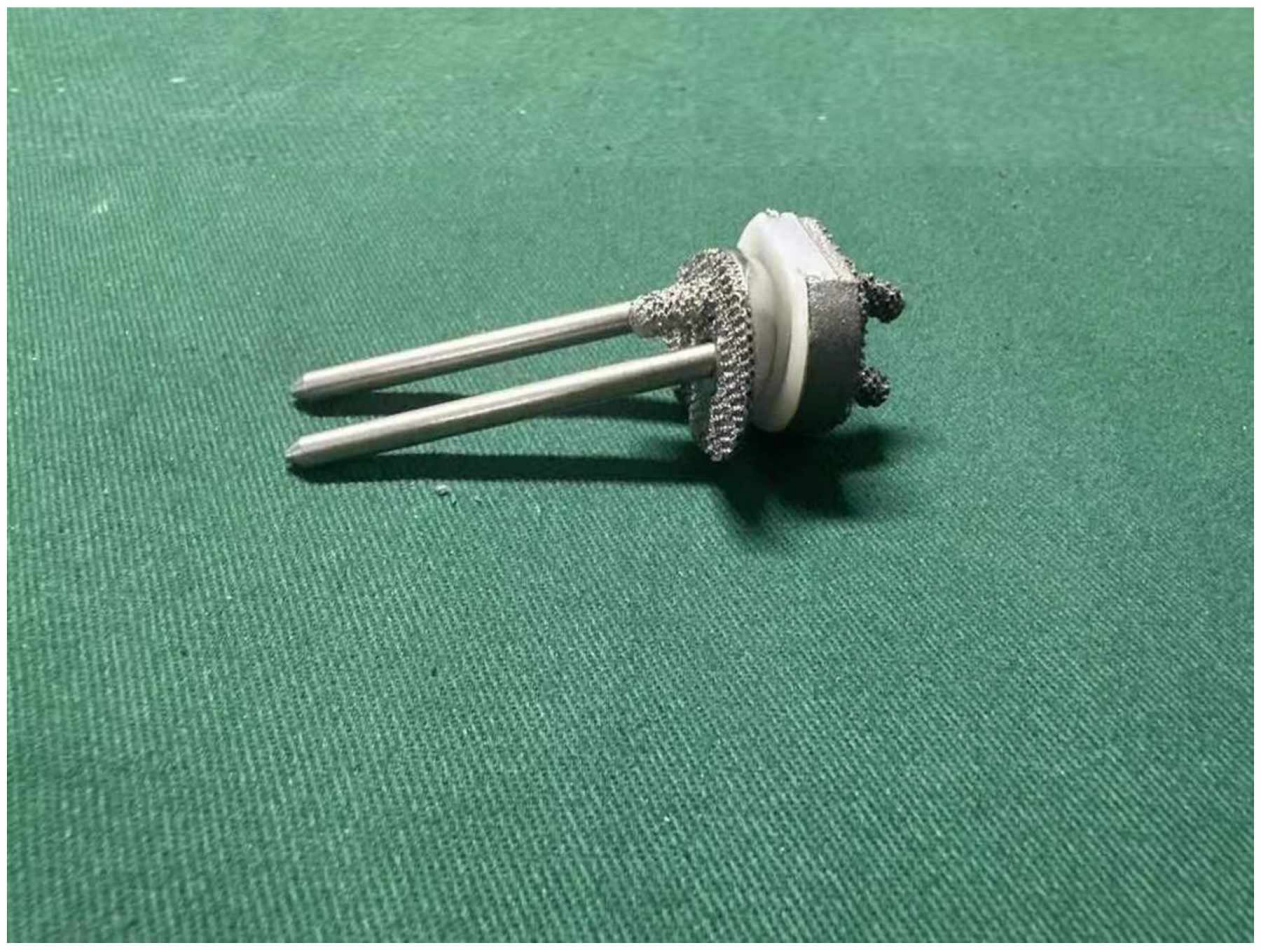
Individualized 3D-printed talonavicular prosthesis. Photograph of the patient-specific talar and navicular components with porous bone-contacting surfaces designed to promote osseointegration.

### Surgical procedure

Following induction of general anesthesia combined with a popliteal sciatic nerve block, the patient was positioned supine. A pneumatic tourniquet was applied at the proximal thigh and inflated to 200 mmHg. A 15-cm longitudinal incision was made along the dorsal midline of the right foot, extending distally from the ankle joint toward the first and second toes. The interval between the extensor hallucis longus and tibialis anterior was carefully dissected to enter the talonavicular joint capsule while protecting the neurovascular structures. Upon exposure, a bony talonavicular coalition was identified. Based on the patient's unique bony morphology, a patient-specific osteotomy guide was designed using anatomical landmarks surrounding the coalition site. Prominent and recessed bony contours around the lesion were used as reference points to ensure accurate intraoperative positioning and fixation of the guide. Individualized drilling guides and prosthetic trial components were designed to facilitate implant preparation and placement. Before implantation, trial fitting was performed using customized molds to verify implant positioning and ensure complete seating without protrusion beyond the planned boundaries.

The patient-specific osteotomy guide was inserted and temporarily secured with Kirschner wires. Osteotomy was performed, after which the guide plate and resected bone were removed. Trial locators for the talar and navicular components were sequentially positioned, temporarily fixed with Kirschner wires after removing the trial locators and wires, the talar component of the prosthesis was implanted with satisfactory fixation, followed by the navicular component. The prosthesis demonstrated stable fixation with satisfactory range of motion at the talonavicular joint. Following removal of the trial components, the definitive talar and navicular prosthetic components were implanted, achieving stable fixation. Intraoperative assessment demonstrated satisfactory joint stability and range of motion.

Adjunctive procedures were subsequently performed to address the associated soft-tissue imbalance and hindfoot deformity, including tibialis posterior tendon tightening (16), peroneus brevis Z-lengthening (17), and calcaneal osteotomy (18). The osteotomy site was distracted by 10 mm, followed by implantation of combined autologous and allogeneic bone grafts and plate fixation. Intraoperative fluoroscopy confirmed satisfactory deformity correction, restoration of the medial arch, and appropriate prosthesis positioning. The wound was closed by layers, and vacuum sealing drainage was applied. The total operative time was 90 min, with an estimated intraoperative blood loss of 20 mL. No blood transfusion was required, and no intraoperative complications occurred.

### Follow-up and outcomes

At the 24-month follow-up, the patient reported complete midfoot pain relief and marked improvement in daily activities. Postoperatively, foot appearance and talonavicular joint mobility were satisfactory (Figure 5).

**Figure 5:**
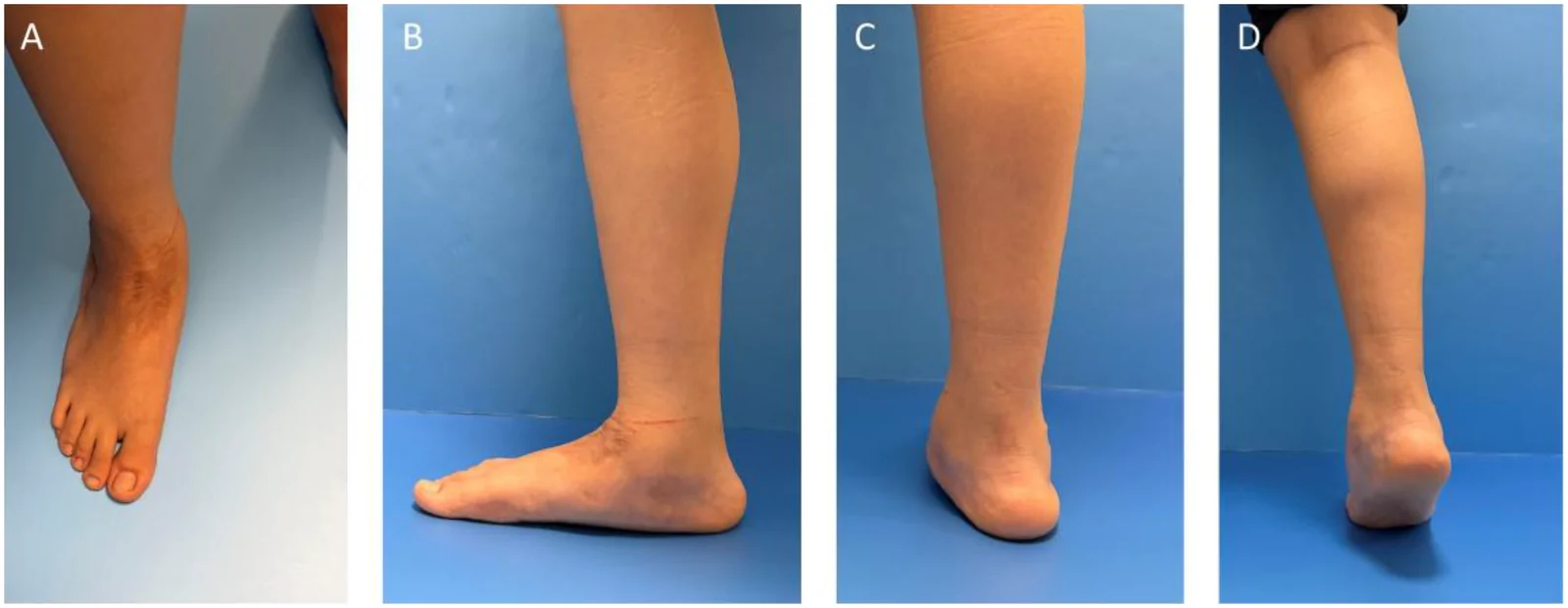
Postoperative photographs of the right foot at 24-months follow-up. **(A,B)** Anteromedial and lateral views demonstrating improvement of the medial longitudinal arch. **(C)** Posterior view showing a reduced hindfoot valgus deformity. **(D)** Heel-rise test indicating improved varus strength.

Clinical assessment demonstrated progressive improvement in pain control and functional capacity. At final follow-up, inversion and eversion were both 15°, with 15° of pronation and supination, indicating satisfactory joint motion (Table 1). The VAS score decreased from 8 preoperatively to 0, while the AOFAS score improved from 64 to 98.

**Table 1:** Patient's clinical and radiological outcomes at serial follow-ups.

Parameter	Preoperation	6 months	12 months	24 months
VAS	8	3	1	0
AOFAS	64	90	95	98
Inversion (°)	10	15	15	15
Eversion (°)	20	15	15	15
Pronation/supination (°)	5	15	15	15
Pitch's angle(°)	1.9	5.3	7.5	9.7
Bohler's angle (°)	6.3	8.9	12.2	16.0
Meary's angle (°)	19.2	18.9	19.7	21.4
Talonavicular coverage angle (°)	4.6	2.1	2.2	2.3
Hindfoot alignment angle(°)	29.5	17.6	10.3	8.8

Radiographic assessment confirmed maintained talonavicular joint congruency and appropriate prosthesis alignment without evidence of subsidence, loosening, osteolysis, or adjacent joint degeneration. At final follow-up, the talonavicular coverage angle was 2.3°. Weight-bearing lateral radiographs demonstrated restoration of the medial longitudinal arch, with a Pitch's angle of 9.7°, Bohler's angle of 16.0°, and Meary's angle of 21.4°. The hindfoot alignment view confirmed correction of valgus deformity, with a hindfoot alignment angle of 8.8° (Figure 6). Radiographic parameters were independently measured by the operating surgeon and another surgeon using miPlatform (v1.3.8.2970; Haiyi Medical Information Technology, Beijing, China). Any discrepancies were resolved by consensus.

**Figure 6:**
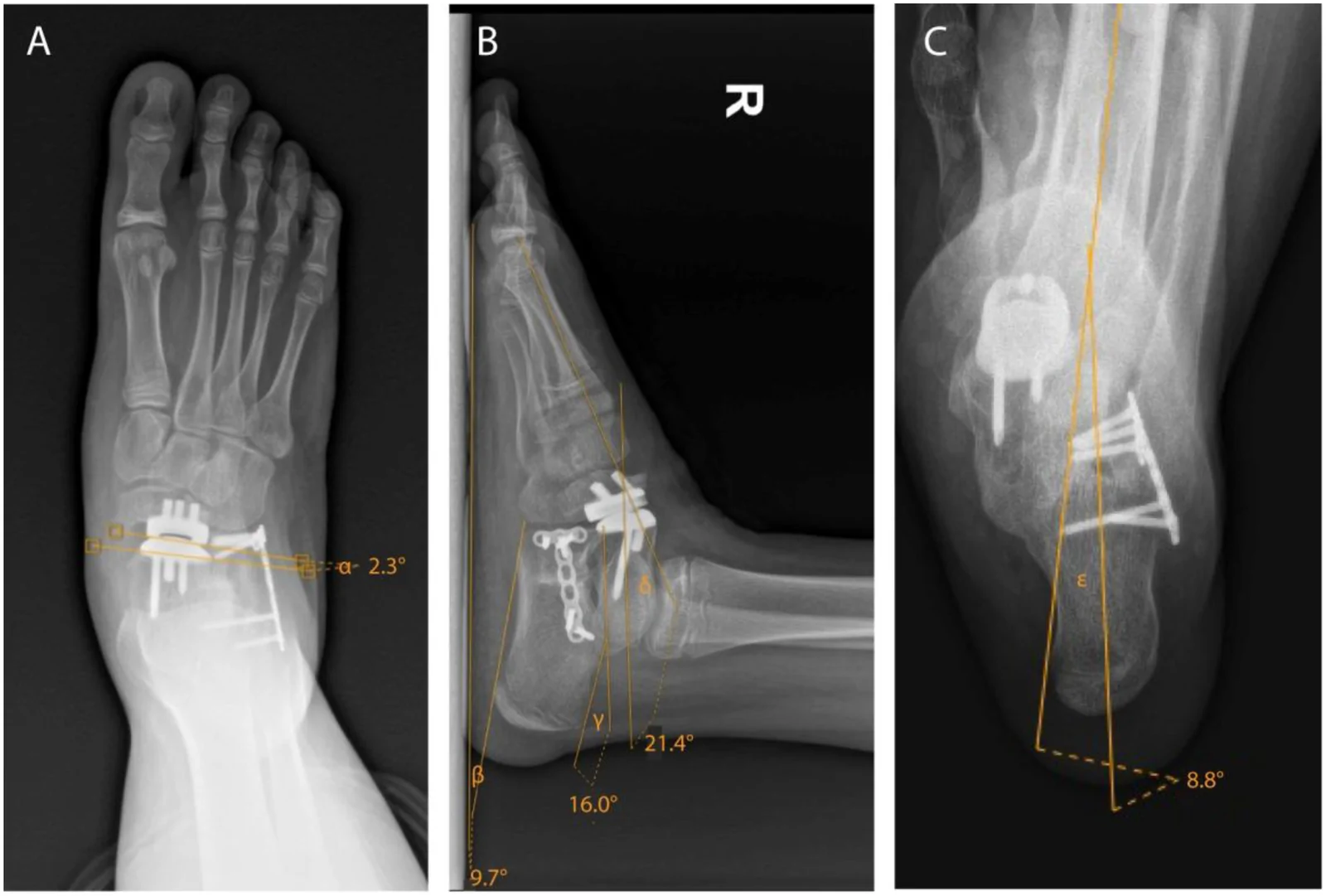
Radiographic evaluation of the right foot at 24-months follow-up. **(A)** Weight-bearing anteroposterior radiograph demonstrating satisfactory talonavicular joint congruency. *α*: Talonavicular coverage angle 2.3°.**(B)** Weight-bearing lateral radiograph showing restoration of the medial longitudinal arch. *β*: Pitch's angle 9.7°, *γ*: Bohler's angle 16.0°, *δ*: Meary's angle 21.4°. **(C)** Hindfoot alignment view confirming correction of the hindfoot valgus. *ε*: Hindfoot alignment angle 8.8°.

## Discussion

Talonavicular joint exhibited the greatest rotational mobility among tarsal joints (19). After arthrodesis, subtalar joint motion decreases by 10%–46%, resulting in significant alterations in joint kinematics (20)**.** Talonavicular arthrodesis sacrifices hindfoot mobility in exchange for stability. It also increases mechanical stress on adjacent joints, thereby accelerating secondary degenerative changes (21). Given the extensive facet involvement, resection alone would compromise medial column support, leading to midfoot instability and arch collapse. A 3D-printed prosthesis was therefore used to reconstruct native midfoot anatomy and ensure precise talonavicular congruency and maintain the joint in an optimal functional position.

Tarsal coalition with substantial flatfoot deformity represents a complex three-dimensional pathology that may require correction beyond coalition treatment alone. Tanghe et al. emphasized that, in patients with talocalcaneal coalition and concomitant rigid flatfoot, coalition resection alone may restore subtalar motion but does not correct the associated deformity, and therefore incorporated peroneus brevis Z-lengthening and calcaneal lateral column lengthening into the reconstructive procedure (17). Lateral column lengthening restores lateral column length and corrects forefoot abduction and hindfoot valgus while improving the medial arch and talonavicular coverage (22, 23). Coalition-specific studies have similarly demonstrated favorable radiographic and clinical outcomes after Evans-type lateral column lengthening in talonavicular and talocalcaneal coalition (24). Patel et al. reported a pediatric patient with isolated symptomatic talonavicular coalition treated with modified Evans lateral column lengthening and iliac crest autografting, with sustained pain relief, early graft incorporation, and maintained alignment at 3 years. Similarly, Shang et al. demonstrated that Evans lateral column lengthening effectively corrected hindfoot valgus and associated deformity in 13 feet with talocalcaneal coalition, improving AOFAS scores from 46.5 to 90.7 and VAS scores from 6.54 to 1.85, with significant improvements in talonavicular coverage(11.00°), calcaneal pitch(18.62°), and hindfoot valgus(4.92°). In our patient, calcaneal osteotomy was therefore incorporated into comprehensive reconstruction. At 24 months, VAS decreased from 8 to 0 and AOFAS increased from 64 to 98, with improved talonavicular coverage (4.6° to 2.3°), calcaneal pitch (1.9° to 9.7°), and hindfoot alignment (29.5° to 8.8°). Meary's angle showed a slight increase from 19.2° to 21.4°, which may indicate a tendency toward recurrent sagittal-plane deformity.

Soft-tissue balancing may further support correction after bony reconstruction. Peroneus brevis Z-lengthening facilitates lateral soft-tissue balancing, whereas tibialis posterior tendon tightening restores medial tension and dynamic arch support (25, 26). Because these procedures are typically performed as components of multicomponent reconstruction, their individual effects are difficult to isolate. Shih et al. reported improved clinical and radiographic outcomes after flatfoot reconstruction incorporating lateral column lengthening and posterior tibial tendon procedures (27). Similarly, our patient underwent combined tibialis posterior tendon tightening, peroneus brevis Z-plasty, calcaneal osteotomy, and patient-specific TNJR, resulting in improvements in pain, function, talonavicular coverage, calcaneal pitch, and hindfoot alignment. Importantly, unlike conventional coalition reconstruction, this strategy also reconstructed the diseased talonavicular articulation while preserving measurable joint motion, with 15° of inversion/eversion and 15° of pronation/supination at 24 months. Thus, our approach extends conventional deformity correction toward a patient-specific, joint-preserving reconstructive strategy.

Despite the favorable 24-month outcome, the long-term performance of individualized TNJR in this skeletally immature patient remains uncertain. Potential risks include bearing wear, loosening, osteolysis, mechanical failure, and revision surgery, while the effects of the implant on future foot growth remain unknown. Although no radiographic complications were observed, longer-term follow-up is warranted to assess implant durability and growth-related changes, particularly the potential for recurrent or progressive medial longitudinal arch collapse.

## Conclusion

This technique may represent a promising motion-preserving option for carefully selected patients with congenital talonavicular coalition, although further studies are required to determine its long-term outcomes and broader clinical applicability.

## Data Availability

The original contributions presented in the study are included in the article/Supplementary Material, further inquiries can be directed to the corresponding authors.
